# Co-carriage of *Staphylococcus aureus* and *Streptococcus pneumoniae* among children younger than 2 years of age in a rural population in Pakistan

**DOI:** 10.1016/j.cegh.2023.101293

**Published:** 2023

**Authors:** Shahira Shahid, Muhammad Imran Nisar, Fyezah Jehan, Sheraz Ahmed, Furqan Kabir, Aneeta Hotwani, Sahrish Muneer, Muhammad Farrukh Qazi, Sajid Muhammad, Asad Ali, Anita K.M. Zaidi, Najeeha T. Iqbal

**Affiliations:** a Department of Pediatric and Child Health, Aga Khan University, Karachi, Pakistan; b Bill & Melinda Gates Foundation, Seattle, WA, USA

**Keywords:** Nasopharyngeal carriage, Pakistan, *Streptococcus pneumoniae*, *Staphylococcus aureus*, MRSA, Children

## Abstract

**Objectives:**

*Staphylococcus aureus* and *Streptococcus pneumoniae* are common colonizers of the human nasopharynx. In this study, we describe *S. aureus* nasopharyngeal carriage and evaluate its association with *S. pneumoniae* carriage post-10-valent pneumococcal conjugate vaccine (PCV10) introduction in Pakistan.

**Methods:**

A serial cross-sectional study was undertaken from 2014 to 2018, children <2 years were randomly selected, and nasopharyngeal swabs were collected using standard WHO guidelines. *S. aureus* and *S. pneumoniae* isolates were identified using standard methods and tested for antimicrobial susceptibility by the standard Kirby-Bauer disk-diffusion method as per Clinical & Laboratory Standards Institute (CLSI) recommendations. Regression analysis was used to determine predictors associated with *S. aureus* carriage.

**Results:**

We enrolled 3140 children. *S. aureus* carriage prevalence was 5.6% (176/3140), and 50.1% (81/176) of the isolates were methicillin-resistant *S. aureus* (MRSA). *S. aureus* carriage was higher in the absence of pneumococcus compared to isolates in which pneumococcus was present (7.5% vs 5.0%). *S. aureus* carriage was negatively associated with pneumococcal carriage, being in 3rd and 4th year of enrollment, and vaccination with two and three PCV10 doses, in addition, fast breathing, ≥2 outpatients visits, and rainy season were positively associated. The following resistance rates were observed: 98.9% for penicillin, 74.4% for fusidic acid, and 23.3% for gentamicin, 10.2% for erythromycin, and 8.5% for cotrimoxazole. All isolates were susceptible to amikacin.

**Conclusions:**

Overall *S. aureus* carriage prevalence was low, PCV10 vaccine was protective against the carriage. The proportion of MRSA carriage and antimicrobial resistance was high in this community warranting continuous monitoring for invasive infections.

## 1 Introduction

*Staphylococcus aureus* is an opportunistic pathogen commonly found in community and hospital settings. It can cause infections of the skin, soft tissue, and bone, pneumonia, and bacteremia.^1^ In 2019*, S. aureus* was responsible for more than 250,000 deaths due to antimicrobial-resistant infections worldwide.^2^ *S aureus* is a common commensal of the skin, pharynx, and groin. It is mostly carried in the anterior nares of the nose which act as reservoirs for transmission to extra-nasal sites.^3^ Carriage acquisition occurs early in life, approximately 70% of newborns carry *S. aureus*. Nasal carriage rates decline to approximately 21% by 6 months of age.^4^ In the nasopharyngeal niche, *S. aureus* competes for nutrients and attachment sites with other co-resident microflora such as *Streptococcus pneumoniae* (pneumococcus)*, Moraxella catarrhalis, Haemophilus influenzae, Neisseria meningitidis,* and various other hemolytic *streptococci* and these interactions may either promote or inhibit its growth in the nasopharynx.^5^ Against the backdrop of increasing multidrug resistance and high carriage rates in children, the interaction between *S. aureus* and pneumococcus is of significant concern since both have a high invasive disease-causing potential.^1^^,^^6^ Pneumococcal carriage is found in as high as 80% of children, particularly in low-and middle-income countries.^6^ Pneumococcal conjugate vaccines (PCVs) have proven to be effective in preventing invasive pneumococcal disease in young children.^7^^,^^8^ However, there are currently no vaccines against diseases caused by *S. aureus*. PCVs alter the microflora dynamics in the nasal passages, by eliminating the vaccine-type serotypes and this may cause a concurrent rise in *S. aureus* carriage.^9^ Several epidemiological studies have demonstrated lower pneumococcal carriage rates in the presence of *S. aureus* nasal carriage.^10^, ^11^, ^12^ It is evident that colonization with pneumococci inversely affects the growth of *S. aureus* in the nasopharynx through the production of H_2_O_2_ and bacteriocins.^13^^,^^14^ This selective pressure could also promote a rise in drug-resistant *S. aureus* carriage. Thus, it is important to monitor the changing epidemiology of pneumococci and *S. aureus* in the post-vaccine era.

Pakistan was the first country in South Asia to introduce the ten-valent PCV (PCV10) in its Expanded Program on Immunization (EPI) in early 2013.^15^ It is given in a schedule of three doses at 6, 10 and 14 weeks of life and no catchup immunization (3 + 0 schedule).^16^ We performed a study to investigate the direct and indirect effects of PCV10 introduction on pneumococcal carriage for which we collected nasopharyngeal swabs from 3140 children over a period of four years from 2014-2018.^17^ Here, we use the available data to study the epidemiology and risk factors for *S. aureus* nasopharyngeal carriage in the same cohort and evaluate its association with *S. pneumoniae* carriage. In addition, we describe the antibiotic susceptibility patterns in *S. aureus* carriage.

## 2 Materials and methods

### 2.1 Study design and setting

We performed serial cross-sectional surveys from October 2014 to September 2018 in two union councils (Khyber and Shah Alam Shah Jee Wasi) of Matiari in Sindh, Pakistan. Matiari is a rural district having a total population of around 88,739. It is located around 180 km away from Karachi. A background demographic surveillance system (DSS) in the two union councils provided us a framework from which we randomly selected 15 age-eligible children every week. Trained data collectors performed household visits to enroll children aged less than 2 years. Each child was only enrolled once in the study after obtaining informed consent from their legal guardian. The vaccination history of the child was collected as a combination of caregiver reported or/and card-verified (where available). Children with nose and throat abnormalities or with a serious illness requiring hospitalization were excluded from the study. Data on household demographics, recent clinical history including hospitalization and outpatient visits, exposure to household smoke and indoor air pollution was collected by study personnel on smartphones. A brief clinical exam including measurement of fever, respiratory rate, and observation for chest wall indrawing was also done.

### 2.2 Nasopharyngeal swab collection

Nasopharyngeal specimens were collected and transported in Skim-milk tryptone glucose glycerol (STGG) media at 2–8 °C from the field site to the Infectious Disease Research Laboratory (IDRL) in Karachi within 8 h of collection using established World Health Organization's (WHO) consensus methods.^18^

### 2.3 Laboratory testing

The samples were vortexed for 10–20 s and then frozen at −80 °C in an upright position till further processing. For the isolation of *S. aureus* colonies, the samples were thawed, vortexed and 200 μl of the sample was added to a mixture of 1 ml rabbit serum, 5 ml Todd Hewitt broth with 0.5% yeast extract and incubated for 6 h at 37 °C. After this, a loopful (10 μl) of the suspension was streaked on both sheep blood agar and colistin-nalidixic-acid-agar plates and incubated for 18–24 h at 37 °C in a CO_2_‐incubator. Small-to-large yellow colonies surrounded by zones of clear beta-hemolysis were seen. Gram staining showed gram-positive cocci in clusters under an oil immersion lens. Further confirmation of the bacteria was done using biochemical tests including coagulase testing (rabbit plasma with EDTA), growth on mannitol salt agar, hemolytic activity on 5% SBA along with DNase, and phenolphthalein phosphate activity (PPA).

For the identification and isolation of pneumococcus, batches of 20–40 samples were thawed, vortexed and 200 μl of the sample was added to a mixture of 1 ml rabbit serum, 5 ml Todd Hewitt Broth with 0.5% yeast extract and incubated for 6 h at 37 °C. After this, one loop full (10 μl) was inoculated onto bilayer sheep blood and colistin-nalidixic-acid-agar. After 18–24 h, the plates were examined for the appearance of alpha-hemolytic colonies and optochin sensitivity and bile solubility tests were performed. Serotypes were deduced using the published sequential multiplex PCR assay and further confirmed by monoplex PCR.^19^^,^^20^ Detailed methods for pneumococcal identification and isolation are described previously.^17^^,^^21^

### 2.4 Antimicrobial susceptibility testing

Isolates were tested for antimicrobial susceptibility by standard Kirby-Bauer disk-diffusion method on Mueller-Hinton Agar (MHA) with 5% sheep blood agar as per the Clinical & Laboratory Standards Institute (CLSI) recommendations.^22^ The antimicrobials tested were penicillin (10 IU), chloramphenicol (30 μg), erythromycin (15 μg), cotrimoxazole (1.25/23.75 μg), tetracycline (30 μg), cefoxitin (30 μg), fusidic Acid (10 μg), ciprofloxacin (5 μg), clindamycin (2 μg), and amikacin (30 μg) and gentamicin (10 μg).

For isolates that tested erythromycin resistant, and clindamycin susceptible or intermediate, inducible clindamycin resistance was tested by D-zone test. Methicillin resistant *S. aureus* (MRSA) were screened using cefoxitin (30 μg) discs by the disc diffusion technique. Cefoxitin zones of inhibition greater than or equal to 22 mm and less than 22 mm were considered phenotypically to indicate methicillin-sensitive *S. aureus* (MSSA) and MRSA, respectively. The results were interpreted according to the Clinical and Laboratory Standards Institute (CLSI) guidelines.^22^ *Methicillin sensitive S. aureus* (MSSA) ATCC 29213 and *methicillin resistant S. aureus* (MRSA) ATCC 33591 were used as quality controls.

### 2.5 Sample size estimation

Our sample size was calculated to detect a 50% decline in pneumococcal carriage rate from a pre-introduction carriage rate of 26.7%–13.35% in the unvaccinated in the fourth year of the study assuming 80% power and 10% of the sample unvaccinated (0 dose received) in the 4th year of the study, so we needed to enroll a total of 747 children per year. We decided to enroll 15 children per week for an approximate sample size of 780 children per year.

### 2.6 Statistical analyses

Pneumococcal vaccine type (VT) carriage was defined as isolation of any of the 10 serotypes included in PCV10 (serotypes 1, 4, 5, 6B, 7F, 9V, 14, 18C, 19F, 23F). Non-vaccine type (NVT) carriage was defined as the presence of all other pneumococci including the non-typeables. A study year ran from October to the September of the next year. Carriage prevalence was calculated out of the total samples collected. To perform a seasonal analysis, we defined rainy months in Matiari as June, July, August and September, and the remaining months as dry months. Children who received all three PCV10 doses were classified as fully vaccinated whereas those who received no dose were unvaccinated. We describe carriage rates of pneumococcus and *S. aureus* by the number of doses of the vaccine received and the study year. Logistic regression analysis was performed to identify predictors of colonization with *S. aureus* in addition, we also identified predictors for MRSA carriage in the population. For model building, all variables with a p-value less than 0.25 in the bivariate analysis were used to build a multivariable model. A backward selection procedure was used to derive a parsimonious model for retaining only variables significant at a p-value ≤0.05. All analysis was performed using STATA version 15.0.

### 2.7 Ethics

This study was performed in line with the principles of the Declaration of Helsinki. Approval was granted by Aga Khan University's Ethical Review Committee (3181-Ped-ERC-14). Written informed consent was obtained from all legal guardians before commencing enrollment.

## 3 Results

### 3.1 Sociodemographic characteristics of the study population

We approached a total of 4181 households during the 4-year study period from which 3140 children under the age of 2 years, meeting our study criterion were enrolled after obtaining a written informed consent from the primary care giver. Figure S1 describes the reasons for non-enrolment in the study. We enrolled 3140 children during the study period. Their mean age was 10.5 months, 50.3% were male, a majority of their primary caretakers 2596 (82.7%) and half of the primary wage earners, 1671 (53.2%) received no education at all; median crowding index (no. of people/no. of rooms) was 5.5 (IQR 4–7). History of cough, runny nose and fever in the past two weeks was common. At the time of enrollment, 6% of the children had fever, 7% had tachypnea and 2.4% had chest wall indrawing. The proportion of fully vaccinated children (card-verified/verbal) was 57.6% whereas 18.7% of the children were unvaccinated. Socio-demographic characteristics are further described in supplemental Table s1.

### 3.2 Carriage prevalence

**Fig. 1:**
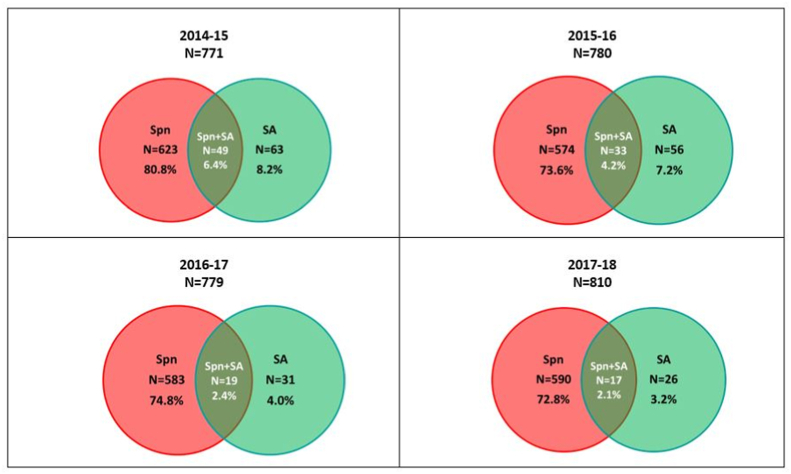
Nasopharyngeal carriage rates of *S. aureus*, pneumococcus and co-carriage from 2014 to 2018 in children less than 2 years of age, n = 3140.

**Fig. 2:**
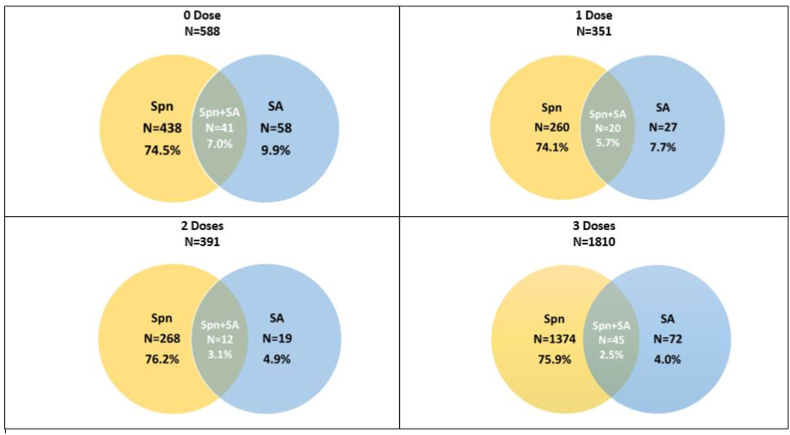
Nasopharyngeal carriage rates of *S. aureus*, pneumococcus and co-carriage according to the number of PCV10 doses received by the children less than 2 years of age, n = 3140.

*S. aureus* was detected in 5.6% (176/3140; 95% CI 4.8–6.5) of the study population. Out of these, 50.6% were methicillin resistant *S. aureus* (MRSA) (Table 1). In the same cohort, carriage of pneumococcus was 75% (2370/3140; 95% CI 74.0–77.0).^17^ Fig. S2 describes the ten most prevalent Vaccine-Type and NVT serotype distribution over the years (2014–2018). *S. aureus* carriage was higher in the absence of pneumococcus compared to isolates in which pneumococcus was present (7.5% vs 5.0%). *S. aureus* carriage was higher during the rainy season than the dry season (8.0% vs 4.4%). The carriage of *S. aureus* declined over the study period from 8.2% (95% CI 6.3–10.3) in 2014 to 3.2% (95% CI 2.1–4.7) in 2018. One-hundred and eighteen, 3.8% (95% CI 3.1–4.5) children had cultures positive for both *S. aureus* and pneumococcus. Co-carriage prevalence declined from 6.4% (95% CI 6.7–8.3) to 2.1% (95% CI 1.2–3.3) during 2014–2018 (Fig. 1). Co-carriage with VT serotypes was 0.7% (22/3140), and with NVT serotypes was 3.0% (96/3140). *S aureus* carriage declined significantly in the fully vaccinated compared to the unvaccinated children 4.0% (95% CI 3.1–5.0) vs 9.9% (95% CI 7.6–12.6) (Table 2 and Fig. 2).

**Table 1:** Carriage rates of *Staphylococcus aureus* from 2014 to 2018 among children less than 2 years of age, n = 3140.

A		2014–15	2015–16	2016–17	2017–18	Total
		(N = 771)	(N = 780)	(N = 779)	(N = 810)	(N = 3140)
		%CI (95%)	%CI (95%)	%CI (95%)	%CI (95%)	%CI (95%)
	Vaccine coverage (3 doses)	41.0	54.6	66.0	68.4	57.6
		(37.5–44.6)	(51.0–58.2)	(62.5–69.3)	(65.1–71.6)	(55.9–59.4)
B	No. of *S. aureus* isolates	63	56	31	26	176
C	*S. aureus* prevalence (B/A)	8.2	7.2	4.0	3.2	5.6
		(6.3,10.3)	(5.5,9.2)	(2.7,5.6)	(2.1,4.7)	(4.8,6.5)
D	No. of pneumococcal isolates	623	574	583	590	2370
E	Pneumococcus prevalence (D/A)	80.8	73.6	74.8	72.8	75.5
		(77.8,83.5)	(70.3,76.7)	(71.6,77.9)	(69.6,75.9)	(73.9,77)
F	No. of isolates where both *S. aureus* and pneumococcus are present	49	33	19	17	118
G	*S. aureus* + pneumococcus prevalence (F/A)	6.4	4.2	2.4	2.1	3.8
		(4.7,8.3)	(2.9,5.9)	(1.5,3.8)	(1.2,3.3)	(3.1,4.5)
H	Carriage of pneumococcus among *S. aureus* isolates (F/B)	77.8	58.9	61.3	65.4	65.9
		(65.5,87.3)	(45.0,71.9)	(42.2,78.2)	(44.3,82.8)	(58.5,72.8)
I	Carriage of *S. aureus* among pneumococcus isolates (F/D)	7.9	5.7	3.3	2.9	5.0
		(5.9,10.3)	(4.0,8.0)	(2.0,5.0)	(1.7,4.6)	(4.1,5.9)

**Table 2:** Nasopharyngeal carriage rates of *Staphylococcus aureus* according to the number of PCV10 doses received by children younger than 2 years of age, n = 3140.

A		0 Dose	1 Dose	2 Dose	3 Dose
		(N = 588)	(N = 351)	(N = 391)	(N = 1810)
		%CI (95%)	%CI (95%)	%CI (95%)	%CI (95%)
B	No. of *S. aureus* isolates	58	27	19	72
C	*S. aureus* prevalence (B/A)	9.9	7.7	4.9	4.0
		(7.6,12.6)	(5.1,11)	(3,7.5)	(3.1,5)
D	No. of pneumococcal isolates	438	260	298	1374
E	Pneumococcus prevalence (D/A)	74.5	74.1	76.2	75.9
		(70.8,78)	(69.2,78.6)	(71.7,80.4)	(73.9,77.9)
F	No. of isolates where both *S. aureus* and pneumococcus are present	41	20	12	45
G	*S. aureus* + pneumococcus prevalence (F/A)	7.0	5.7	3.1	2.5
		(5,9.3)	(3.5,8.7)	(1.6,5.3)	(1.8,3.3)
H	Carriage of pneumococcus among *S. aureus* isolates (F/B)	70.7	74.1	63.2	62.5
		(57.3,81.9)	(53.7,88.9)	(38.4,83.7)	(50.3,73.6)
I	Carriage of *S. aureus* among pneumococcus isolates (F/D)	9.4	7.7	4.0	3.3
		(6.8,12.5)	(4.8,11.6)	(2.1,6.9)	(2.4,4.4)

### 3.3 Predictors of *S. aureus* carriage

Table 3 describes the bivariate and multivariate analysis for the predictors of *S. aureus* carriage. The final model showed a negative association with pneumococcal carriage (aOR 0.7 95%CI 0.5–0.9), crowding index (aOR 0.9 95%CI 0.9–1.0), cough (aOR 0.6 95%CI 0.4–0.9), being enrolled in the third (aOR 0.5 95%CI 0.3–0.8) and fourth year of study (aOR 0.4 95%CI 0.3–0.8), having received two (aOR 0.5 95%CI 0.3–0.9) and three PCV10 doses (aOR 0.4 95%CI 0.3–0.6). *S. aureus* carriage showed a positive association in children with fast breathing on clinical exam (aOR 1.7 95%CI 1.1–2.9), two or more outpatients visits in the previous month (aOR 1.6 95%CI 1.1–2.4), enrollment during the rainy season (aOR 1.7 95%CI 1.2–2.3) (Table 3).

**Table 3:** Predictors of nasopharyngeal carriage of *Staphylococcus aureus* among children less than 2 years of age, n = 3140.

Characteristics	Positive	Negative	OR (95%CI)	aOR (95%CI)
N	176	2964		
	n (%)	n (%)		
**Age group (in months)**				–
0-12	106 (5.1%)	1962 (94.9%)	Ref	
>12-23	70 (6.5%)	1002 (93.5%)	1.3(0.9,1.8)	
**Gender**				
Male	89 (5.6%)	1491 (94.4%)	Ref	–
Female	87 (5.6%)	1473 (94.4%)	1(0.7,1.3)	–
**Primary care taker's education**				
No education	151 (5.8%)	2445 (94.2%)	Ref	–
1–5 years	20 (5.7%)	331 (94.3%)	1(0.6,1.6)	–
6–10 years	3 (2.1%)	137 (97.9%)	0.4(0.1,1.1)	–
>10 years	2 (3.8%)	51 (96.2%)	0.6(0.2,2.6)	–
**Primary wage earner's education**				–
No education	98 (5.9%)	1573 (94.1%)	Ref	
1–5 years	47 (6.2%)	714 (93.8%)	1.1(0.7,1.5)	–
6–10 years	17 (4.1%)	402 (95.9%)	0.7(0.4,1.1)	–
>10 years	14 (4.8%)	275 (95.2%)	0.8(0.5,1.5)	–
**Crowding index, Median (IQR)**	5 (4–7)	5.5 (4–7)	0.9(0.9,1.0)	0.9(0.9,1.0)
**Outpatient visits during the past month**				
None	79 (4.9%)	1532 (95.1%)	Ref	Ref
One	41 (5.1%)	756 (94.9%)	1.1(0.7,1.5)	1.1(0.8,1.7)
Two or more	56 (7.7%)	676 (92.3%)	1.6(1.1,2.3)	1.6(1.1,2.4)
**Hospital admissions during the past year**	5 (5.3%)	89 (94.7%)	0.9(0.4,2.4)	–
**Child exposure to ETS**	66 (5.9%)	1050 (94.1%)	1.1(0.8,1.5)	
**Fuel used for cooking**				–
Natural Gas	18 (3.7%)	467 (96.3%)	Ref	–
Others	158 (6.0%)	2497 (94.0%)	1.6(1.0,2.7)	–
**Child exposure to smoke during cooking**	75 (4.4%)	1614 (95.6%)	0.6(0.5,0.8)	–
Symptoms during last two weeks*				
Runny Nose	78 (4.9%)	1506 (95.1%)	0.7(0.6,1.0)	–
Cough	58 (4.8%)	1162 (95.2%)	0.7(0.5,1.0)	0.6(0.4,0.9)
Fever	84 (5.7%)	1379 (94.3%)	1(0.8,1.4)	–
Fast breathing	5 (6.3%)	74 (93.7%)	1.1(0.4,2.8)	–
Difficulty in breathing	34 (5.6%)	578 (94.4%)	1(0.7,1.4)	–
**Signs**				
Lower chest indrawing	6 (8.1%)	68 (91.9%)	1.5(0.6,3.5)	–
Hypothermia	1 (9.1%)	10 (90.9%)	1.6(0.2,12.7)	–
Hyperthermia	6 (3.2%)	181 (96.8%)	0.5(0.2,1.2)	–
Normal	167 (5.8%)	2703 (94.2%)	Ref	–
Tachypnea (as per WHO cutoffs)	21 (9.9%)	192 (90.1%)	1.9(1.2,3.1)	1.7(1.1,2.9)
**Year of enrollment**				
2014/15	63 (8.2%)	708 (91.8%)	Ref	Ref
2015/16	56 (7.2%)	724 (92.8%)	0.9(0.6,1.3)	0.9(0.6,1.4)
2016/17	31 (4.0%)	748 (96.0%)	0.5(0.3,0.7)	0.5(0.3,0.8)
2017/18	26 (3.2%)	784 (96.8%)	0.4(0.2,0.5)	0.4(0.3,0.8)
**Number of PCV10 doses**				
0	58 (9.9%)	530 (90.1%)	Ref	Ref
1	27 (7.7%)	324 (92.3%)	0.8(0.5,1.2)	0.9(0.5,1.4)
2	19 (4.9%)	372 (95.1%)	0.5(0.3,0.8)	0.5(0.3,0.9)
3	72 (4.0%)	1738 (96.0%)	0.4(0.3,0.5)	0.4(0.3,0.6)
**Season**				
Dry	94 (4.4%)	2022 (95.6%)	Ref	Ref
Rainy	82 (8.0%)	942 (92.0%)	1.9(1.4,2.5)	1.7(1.2,2.3)
**Pneumococcal carriage**				
Yes	118 (5.0%)	2252 (95.0%)	0.6(0.5,0.9)	0.7(0.5,0.9)
No	58 (7.5%)	713 (92.5%)	Ref	Ref
**VT carriage**				
Yes	22 (5.8%)	357 (94.2%)	1.0(0.7,1.6)	–
No	154 (5.6%)	2607 (94.4%)	Ref	–
**NVT carriage**				
Yes	96 (4.8%)	1894 (95.2%)	0.7(0.5,0.9)	–
No	80 (7.0%)	1070 (93.0%)	Ref	–

IQR- Interquartile range, ETS- Environmental Tobacco Smoke, VT-Vaccine type serotypes, NVT-Nonvaccine type serotypes, WHO-World Health Organization *data available for 3068 children.

Tachypnea is defined as children younger than 2 months - Greater than or equal to 60 breaths/min, children aged 2–11 months - Greater than or equal to 50 breaths/min, children aged 12–59 months - Greater than or equal to 40 breaths/min. Hypothermia is defined as the underarm temperature below 35.0 °C (95.0 °F), Hyperthermia is defined as underarm temperature ≥38.0 °C (100.4 °F).

Supplemental Table S2 describes the predictors for MRSA carriage, it was positively associated with primary wage earner education of 6–10 years (aOR 4.0 95%CI 1.2–13.5) and in children with at least one hospital visit in the previous month (aOR 2.7 95%CI 1.3–5.6).

### 3.4 Antimicrobial susceptibility patterns of the isolates

**Fig. 3:**
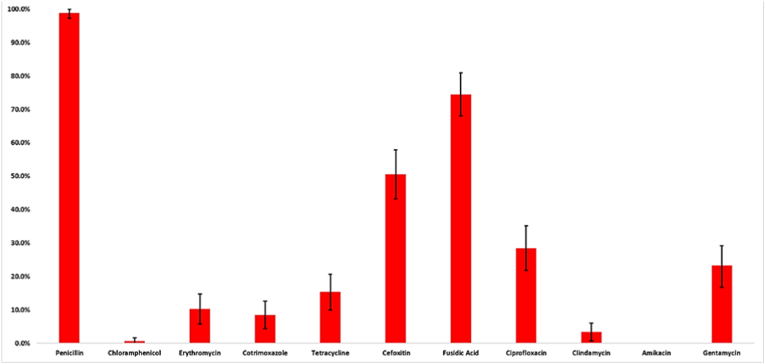
Proportion of *S. aureus* positive isolates which were non-susceptible to various antimicrobial agents (n = 176).

All the 176 isolates were found to be susceptible to amikacin. Non-susceptibility to penicillin was seen in 98.9% (174/176, 95% CI 97.3–100%) of the isolates, 74.4% (131/176, 95% CI 68.0–80.9%) were resistant to fusidic acid, 50.6% (89/176, 95% CI 43.2–58.0%) to cefoxitin, 28.4% (50/176, 95% CI 21.7–31.5%) to ciprofloxacin, 23.3% (41/176, 95% CI 16.8–29.2%) to gentamicin, 15.3% (27/176, 95% CI 10.0–20.7%) were resistant to tetracycline, 10.2% (18/176, 95% CI 5.8–14.7%) were resistant to erythromycin, and 8.5% (15/176, 95% CI 4.4–12.6%) were non-susceptible to cotrimoxazole. However, only 3.4% (6/176, 95% CI 0.7–6.1%) of the isolates were resistant to clindamycin and 0.6% (1/176, 95% CI 0–1.7%) were resistant to chloramphenicol (Fig. 3).

## 4 Discussion

Nasopharyngeal carriage of *S. aureus* declined significantly from 2014 to 2018 in children under 2 years of age. Two-thirds of the time *S. aureus* was accompanied by pneumococcus. Half the *S. aureus* isolates were methicillin resistant (MRSA). *S. aureus* carriage was lower in children who had received 3 doses of PCV10 as compared to unvaccinated children.

*S. aureus* carriage in our study is comparable to some other population-based studies around the world. In a study carried out in India, 6.3% of the children younger than 5 years of age carried *S. aureus* and 3.3% of children aged 17 months in Fiji carried *S. aureus*.^23^^,^^24^ Other countries have reported higher carriage rates. In the pediatric population in eastern Uganda, it was reported to be 19.4%, while in a longitudinal study with repeated sampling in The Gambia, the nasopharyngeal carriage in newborns decreased from 50% to 10% by 9 weeks of age.^25^^,^^26^

*S. aureus* carriage was higher in the absence of pneumococcus compared to isolates in which pneumococcus was present (7.5% vs 5.0%). This inverse association between *S. aureus* and pneumococcal carriage has also been seen in other parts of the world.^25^^,^^27^^,^^28^ A biological explanation for this could be the hydrogen peroxide-mediated killing of *S aureus* by *S. pneumoniae* and a host immune response induced by the pneumococcal pilus against *S. aureus* colonization.^29^, ^30^, ^31^ We observed a negative association between *S. aureus* and NVT pneumococcal carriage. As the NVT carriage increased over time with increasing vaccine coverage, a decline was seen in *S. aureus* carriage. This has been shown in other studies as well.^12^^,^^32^

A surprising finding from the study was that two and three doses of PCV10 were protective against *S. aureus* colonization. The mechanisms mediating this indirect PCV10 effect on non-pneumococcal colonization are not clear. A cross-reactive antibody response or a change in the metabolic activity of commensals could be involved.^33^^,^^34^ In a randomized controlled trial, PCV10 vaccinated children in the 11–13 months age group had lower *S. aureus* nasopharyngeal carriage compared to the PCV7 group.^35^ A decline in the prevalence of major commensals (including *S. aureus*) was seen in PCV7 vaccinated Swiss children aged less than 2 years.^36^ In contrast. Biesbroek et al. found a significant increase in nasopharyngeal microbiome diversity (including *S. aureus*) following PCV7 vaccination in Dutch infants.^37^

Among the predictors for *S. aureus* carriage, symptoms of respiratory tract infection such as tachypnea were positively associated as seen previously.^38^ We saw a positive association between healthcare exposure and both overall carriage and MRSA carriage which has also been confirmed by other studies.^39^^,^^40^ In our study*, S. aureus* carriage had a seasonal pattern, and was positively associated with the rainy season. Two other studies from rural Gambia and Ghana have also reported an increase in *S. aureus* colonization during the rainy season.^41^^,^^42^ We observed a protective effect of the third and final year of enrollment against *S. aureus* when adjusted for all other factors. This could be reflective of a change in the population in terms of hygiene practices, breastfeeding habits, antibiotic use, or other unknown and unmeasured factors. The children may have developed acquired immunity against carriage over time. Bacterial interference in the form of antagonism between *S. aureus* and other bacterial members of the normal flora, such as *S. epidermidis* or *Corynebacterium* could also be responsible for the decline in the carriage over the study year.

Previous studies from Pakistan done between 2005 and 2010 have shown MRSA rates of 12%–28%.^43^ Other LMICs have also reported lower rates ranging from 3.7% to 31%.^26^^,^^44^, ^45^, ^46^ The highest antimicrobial resistance rates in our study were against penicillin and fusidic acid. Previous hospital-based *S. aureus* data also shows high resistance to penicillin.^47^ High resistance rates against fusidic acid render it ineffective against skin and soft tissue infections.

This was a large community-based study leveraging upon an ongoing impact evaluation of the pneumococcal conjugate vaccine in the area. We had certain limitations. We could not collect information on prior antibiotic use in the population which is known to impact the prevalence of MRSA. We could not perform a molecular analysis for strains as no funds were available for this. Our study design did not allow the collection of more than one sample for each child to differentiate between intermittent carriers and non-carriers.

## 5 Conclusion

Overall *S. aureus* carriage prevalence was low, PCV10 vaccine was protective against carriage. The proportion of MRSA carriage and antimicrobial resistance was high in this community warranting continuous monitoring for invasive *S. aureus* infections.

## Author contributions

Conceptualization, Funding acquisition, 282 Methodology and Resources: Asad Ali, Anita K.M. Zaidi, Fyezah Jehan, Muhammad Imran Nisar, Najeeha T. Iqbal; Investigation: Aneeta Hotwani, Furqan Kabir, Sahrish Muneer, Sheraz Ahmed; Data curation: Muhammad Farrukh Qazi, Sajid Muhammad, Sheraz Ahmed; Formal analysis and Visualization: Muhammad Imran Nisar, Muhammad Farrukh Qazi, Sajid Muhammad, Shahira Shahid; Supervision and Project administration: Asad Ali, Aneeta Hotwani, Anita K.M. Zaidi, Furqan Kabir, Fyezah Jehan, Muhammad Imran Nisar, Najeeha T. Iqbal, Sheraz Ahmed. Writing - original draft preparation: all authors; Writing - review and editing: all authors; All authors have read and agreed to the published version of the manuscript.

## Funding

This work was supported by 10.13039/100000865Bill & Melinda Gates Foundation through grant ID #OPP1111303. The authors declare that no funds, grants, or other support were received during the preparation of this manuscript.

## Declaration of competing interest

The authors have no relevant financial or non-financial interests to disclose.
