# Supplementary material for: Co-carriage of Staphylococcus aureus and Streptococcus pneumoniae among children younger than 2 years of age in a rural population in Pakistan
Source: Clin Epidemiol Glob Health. 2023 May-Jun;21:None. doi: 10.1016/j.cegh.2023.101293 (PMC10276771; doi:10.1016/j.cegh.2023.101293)
Supplement: Multimedia component 1 [file mmc1.docx]

**Co-carriage of *Staphylococcus aureus* and *Streptococcus pneumoniae* among children younger than 2 years of age in a rural population in Pakistan**

**Fig S1 Flow of participants in the study**

**
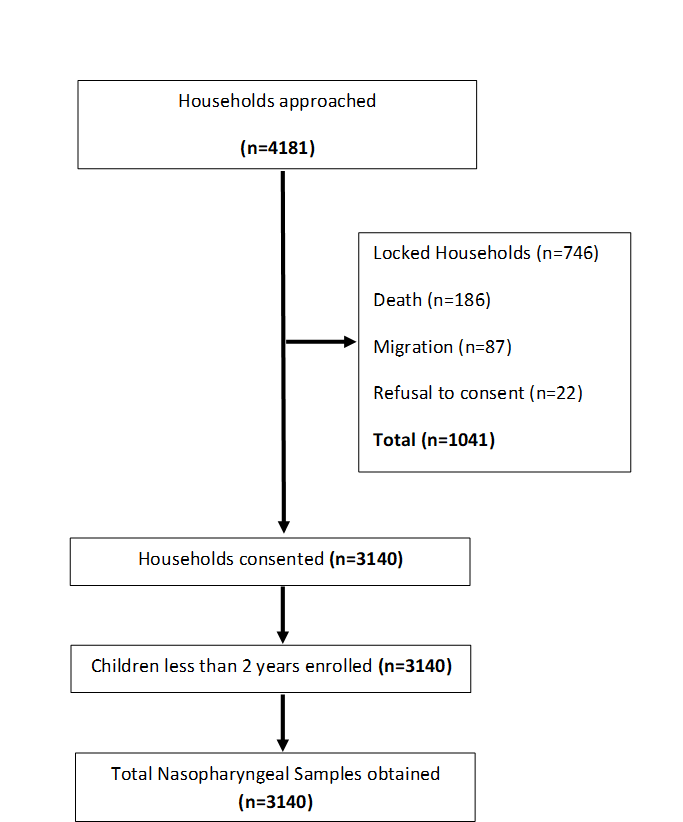
**

**Fig S2 Ten most prevalent Vaccine-Type and NVT serotype distribution over the years (2014–2018)**


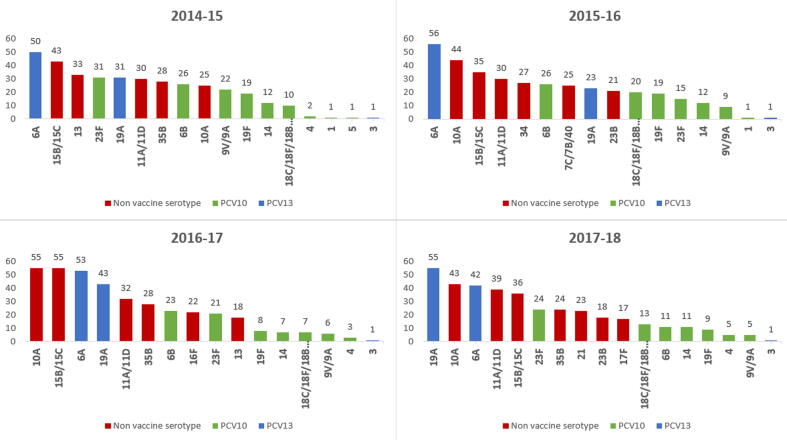


**Table s1: Baseline Characteristics of the study participants**

| **Characteristics** | **N=3,140** |
| --- | --- |
| **Age, months (mean ± SD)** | 10.5 ± 9.5 |
| 0-3 months | 305 (9.7%) |
| 4-11 months | 1,603 (51.1%) |
| 12-23 months | 1,232 (39.2%) |
| **Gender** |  |
| Male | 1,580 (50.3%) |
| Female | 1,560 (49.7%) |
| **Primary caretaker education** |  |
| no education | 2,596 (82.7%) |
| 1-5 years | 351 (11.2%) |
| 6 to 10 years | 140 ( 4.5%) |
| >10 years | 53 ( 1.7%) |
| **Primary wage earner education** |  |
| no education | 1,671 (53.2%) |
| 1-5 years | 761 (24.2%) |
| 6 to 10 years | 419 (13.3%) |
| >10 years | 289 ( 9.2%) |
| **Crowding index, Median (IQR)** | 5.5 (4-7) |
| **Hospital visits in last month** |  |
| None | 1,611 (51.3%) |
| One | 797 (25.4%) |
| Two or more | 732 (23.3%) |
| **Hospital Admissions in last year** |  |
| Yes | 3,046 (97.0%) |
| No | 94 ( 3.0%) |
| **Smoker in household** | 1,116 (35.5%) |
| **Fuel use for cooking** |  |
| Natural Gas | 485 (15.4%) |
| Others | 2,655 (84.6%) |
| **Child exposure to smoke during cooking** | 1,689 (53.8%) |
| **Symptoms during last two weeks, n= 3068** |  |
| Runny Nose | 1,584 (51.6%) |
| Cough | 1,220 (39.8%) |
| Fever | 1,463 (47.7%) |
| Fast breathing | 79 ( 2.6%) |
| Difficulty in breathing | 612 (19.9%) |
| **Signs** |  |
| Lower chest indrawing | 74 ( 2.4%) |
| Hypothermia | 11 ( 0.4%) |
| Hyperthermia | 187 ( 6.1%) |
| Normal | 2,870 (93.5%) |
| Tachypnea (as per WHO cutoffs) | 213 ( 6.9%) |
| **Year of enrollment** |  |
| 2014/15 | 771 (24.6%) |
| 2015/16 | 780 (24.8%) |
| 2016/17 | 779 (24.8%) |
| 2017/18 | 810 (25.8%) |
| **No. of PCV doses** |  |
| 0 | 588 (18.7%) |
| 1 | 351 (11.2%) |
| 2 | 391 (12.5%) |
| 3 | 1,810 (57.6%) |
| **No. of participants enrolled season-wise** |  |
| Dry season | 2,116 (67.4%) |
| Rainy season | 1,024 (32.6%) |
| **Pneumococcus positive** | 2,370 (75.5%) |
| **S. aureus positive** | 176 (5.6%) |
| MRSA, n= 176 | 89 (50.6%) |
| **Co-carriage** | 118 (3.8%) |

**Table s2: Predictors of MRSA carriage**

| **Characteristics** | **Resistant** | **Sensitive** | **OR (95%CI)** | **P-value** | **AOR (95%CI)** | **P-value** |
| --- | --- | --- | --- | --- | --- | --- |
|  | **N= 89** | **N= 87** |  |  |  |  |
| **Age, months** |  |  |  |  |  |  |
| 0-12 months | 52 (49.1%) | 54 (50.9%) | Ref |  | - | - |
| >12-23 months | 37 (52.9%) | 33 (47.1%) | 1.2(0.6,2.1) | 0.49 | - | - |
| **Gender** |  |  |  |  |  |  |
| Male | 47 (53%) | 42 (47%) | Ref |  | - | - |
| Female | 42 (48%) | 45 (52%) | 0.8(0.5,1.5) | 0.548 | - | - |
| **Primary caretaker education** |  |  |  |  |  |  |
| no education | 79 (52.3%) | 72 (47.7%) | Ref |  | - | - |
| 1-5 years | 8 (40.0%) | 12 (60.0%) | 0.6(0.2,1.6) | 0.304 | - | - |
| 6 to 10 years | 1 (33.3%) | 2 (66.7%) | 0.5(0,5.1) | 0.525 | - | - |
| >10 years | 1 (50.0%) | 1 (50.0%) | 0.9(0.1,14.8) | 0.948 | - | - |
| **Primary wage earner education** |  |  |  |  |  |  |
| no education | 42 (43%) | 56 (57%) | Ref |  | Ref | - |
| 1-5 years | 27 (57%) | 20 (43%) | 1.8(0.9,3.6) | 0.101 | 1.8(0.8,3.6) | 0.122 |
| 6 to 10 years | 13 (76%) | 4 (24%) | 4.3(1.3,14.2) | 0.016 | 4.0(1.2,13.5) | 0.024 |
| >10 years | 7 (50%) | 7 (50%) | 1.3(0.4,4.1) | 0.615 | 1.4(0.3,0.9) | 0.554 |
| **Crowding index, Median(IQR)** | 5 (4-7) | 5 (4-7) | 1(0.8,1.1) | 0.51 | - | - |
| **Hospital visits in last month** |  |  |  |  |  |  |
| None | 32 (41%) | 47 (59%) | Ref |  | Ref |  |
| One | 20 (49%) | 21 (51%) | 1.4(0.7,3) | 0.386 | 1.3(0.6,2.9) | 0.451 |
| Two or more | 37 (66%) | 19 (34%) | 2.9(1.4,5.8) | 0.004 | 2.7(1.3,5.6) | 0.007 |
| **Hospital Admissions in last year** |  |  |  |  |  |  |
| Yes | 4 (80.0%) | 1 (20.0%) | 4.0(0.4,36.9) | 0.215 | - | - |
| No | 85 (49.7%) | 86 (50.3%) | Ref |  | - | - |
| **Smoker in household** |  |  |  |  |  |  |
| Yes | 32 (48.5%) | 34 (51.5%) | 0.9(0.5,1.6) | 0.669 | - | - |
| No | 57 (51.8%) | 53 (48.2%) | Ref |  | - | - |
| **Fuel use for cooking** |  |  |  |  |  |  |
| Natural Gas | 10 (55.6%) | 8 (44.4%) | Ref |  | - | - |
| Others | 79 (50.0%) | 79 (50.0%) | 0.8(0.3,2.1) | 0.656 | - | - |
| **Child exposure to smoke during cooking** |  |  |  |  |  |  |
| Yes | 40 (53.3%) | 35 (46.7%) | 1.2(0.7,2.2) | 0.527 | - | - |
| No | 49 (48.5%) | 52 (51.5%) | Ref |  | - | - |
| **Symptoms during last two weeks** |  |  |  |  |  |  |
| **Runny Nose** |  |  |  |  |  |  |
| Yes | 40 (51%) | 38 (49%) | 1.1(0.6,2) | 0.76 | - | - |
| No | 47 (49%) | 49 (51%) | Ref |  | - | - |
| **Cough** |  |  |  |  |  |  |
| Yes | 34 (58.6%) | 24 (41.4%) | 1.7(0.9,3.2) | 0.109 | - | - |
| No | 53 (45.7%) | 63 (54.3%) | Ref |  | - | - |
| **Fever** |  |  |  |  |  |  |
| Yes | 47 (56%) | 37 (44%) | 1.6(0.9,2.9) | 0.13 | - | - |
| No | 40 (44%) | 50 (56%) | Ref |  | - | - |
| **Fast breathing** |  |  |  |  |  |  |
| Yes | 4 (80.0%) | 1 (20.0%) | 4.1(0.5,37.9) | 0.208 | - | - |
| No | 83 (49.1%) | 86 (50.9%) | Ref |  | - | - |
| **Difficulty in breathing** |  |  |  |  |  |  |
| Yes | 17 (50.0%) | 17 (50.0%) | 1(0.5,2.1) | 0.999 | - | - |
| No | 70 (50.0%) | 70 (50.0%) | Ref |  | - | - |
| **Lower chest indrawing** |  |  |  |  |  |  |
| Yes | 4 (66.7%) | 2 (33.3%) | 2(0.4,11.5) | 0.415 | - | - |
| No | 83 (49.4%) | 85 (50.6%) | Ref |  | - | - |
| **Signs** |  |  |  |  |  |  |
| Hypothermia | 0 ( 0.0%) | 1 (100.0%) | - |  | - | - |
| Hyperthermia | 3 (50.0%) | 3 (50.0%) | 1.0(0.2,5.0) | 0.989 | - | - |
| Normal | 84 (50.3%) | 83 (49.7%) | Ref | - | - | - |
| **Tachypnea (as per WHO cutoffs)** |  |  |  |  |  |  |
| Yes | 10 (47.6%) | 11 (52.4%) | 0.9(0.4,2.2) | 0.816 | - | - |
| No | 77 (50.3%) | 76 (49.7%) | Ref |  | - | - |
| **Year of enrollment** |  |  |  |  |  |  |
| 2014/15 | 29 (46%) | 34 (54%) | Ref |  | - | - |
| 2015/16 | 27 (48%) | 29 (52%) | 1.1(0.5,2.2) | 0.812 | - | - |
| 2016/17 | 17 (55%) | 14 (45%) | 1.4(0.6,3.4) | 0.423 | - | - |
| 2017/18 | 16 (62%) | 10 (38%) | 1.9(0.7,4.8) | 0.186 | - | - |
| **No. of PCV doses** |  |  |  |  |  |  |
| 0 | 26 (45%) | 32 (55%) | Ref |  | - | - |
| 1 | 12 (44%) | 15 (56%) | 1(0.4,2.5) | 0.974 | - | - |
| 2 | 11 (58%) | 8 (42%) | 1.7(0.6,4.8) | 0.325 | - | - |
| 3 | 40 (56%) | 32 (44%) | 1.5(0.8,3.1) | 0.225 | - | - |
| **Season** |  |  |  |  |  |  |
| Dry (<1 mm rainfall) | 53 (56%) | 41 (44%) | Ref |  | - |  |
| Wet (≥1 mm rainfall) | 36 (44%) | 46 (56%) | 0.6(0.3,1.1) | 0.099 |  |  |
| **Pneumococcus** |  |  |  |  |  |  |
| Yes | 55 (46.6%) | 63 (53.4%) | 0.6(0.3,1.2) | 0.135 | - | - |
| No | 34 (58.6%) | 24 (41.4%) | Ref |  | - | - |
| **VT carriage** |  |  |  |  |  |  |
| Yes | 11 (50.0%) | 11 (50.0%) | 1.0(0.4,2.4) | 0.955 | - | - |
| No | 78 (50.6%) | 76 (49.4%) | Ref |  | - | - |
| **NVT carriage** |  |  |  |  |  |  |
| Yes | 44 (46%) | 52 (54%) | 0.7(0.4,1.2) | 0.17 | - | - |
| No | 45 (56%) | 35 (44%) | Ref |  | - | - |
